# Commentary: Geographic Variations in the Incidence of Glioblastoma and Prognostic Factors Predictive of Overall Survival in US Adults from 2004–2013

**DOI:** 10.3389/fnagi.2018.00105

**Published:** 2018-04-12

**Authors:** S. M. J. Mortazavi

**Affiliations:** ^1^Diagnostic Imaging Department, Fox Chase Cancer Center, Philadelphia, PA, United States; ^2^Ionizing and Non-ionizing Radiation Protection Research Center (INIRPRC), Shiraz University of Medical Sciences, Shiraz, Iran

**Keywords:** brain cancer, glioblastoma, geographic variations, mobile phones, electromagnetic fields

Xu et al. in their recent article entitled “Geographic Variations in the Incidence of Glioblastoma and Prognostic Factors Predictive of Overall Survival in US Adults from 2004 to 2013” that is published in the Front. Aging Neurosci (Xu et al., 2017) have evaluated 24,262 glioblastoma patients. They showed that the incidence of glioblastoma is dependent on factors such as geographic region and race/ethnicity.

With a relatively short survival rate, glioblastoma is the most common primary brain tumor. As it is unlikely that a single biomarker can effectively detect glioblastoma, scientists have tried to use a novel combination of multiple biomarkers (Popescu et al., 2014). Experiments performed for better understanding of cellular and molecular events involved in glioblastoma pathogenesis, show that the PI3K pathway can be a prime target for treatment of glioblastoma (Cruceru et al., 2013). Despite its strengths, this paper has at least one major shortcoming. Over the past decade, my colleagues and I have studied the health effects of cellular phones and their base stations. We have also shown that some of the papers claiming no link between mobile phone use and cancer have methodological errors and/or shortcomings (Mortazavi et al., 2017a,b, 2018; Mortazavi, 2018). For example, in one of the papers reviewed by our research group, a 400% difference in brain tumors was masked by poor statistical analysis! (Mortazavi, 2018).

The major shortcoming of the paper authored by Xu et al. comes from this point that the authors have not considered substantial evidence that shows a significant association between mobile phone use and brain cancer “*Other potential risk factors like cell phone use, smoking, and environmental exposures have been studied, however, the conclusions were not definitive* (Gomes et al., 2011).” For example, a significant association between mobile and cordless phone use and malignant brain tumors was reported in a case-control study on brain tumors performed by Hardell et al. The authors claimed that the results of their study were in line with this hypothesis that exposure to radiofrequency electromagnetic fields (RF-EMFs) generated by wireless phones can play a significant role in the initiation and promotion of cancer (Hardell et al., 2013). Hardell and Carlberg in their recent report regarding Average Annual Percentage Change (AAPC) of CNS cancers stated “*In summary this register based study showed increasing rates of tumors of unknown type in CNS (D43) with higher rate during 2007–2015. AAPS increased especially in the age group 20–39 years at diagnosis. This may be explained by higher risk for brain tumor in subjects with first use of a wireless phone before the age of 20 years taking a reasonable latency period*” (Hardell and Carlberg, 2017). Moreover, a meta-analysis of 24 studies (26,846 cases, 50,013 controls) also supported this hypothesis that long-term use of mobile phones can be associated with increased risk of intracranial tumors (Bortkiewicz et al., 2017). The findings of this study revealed that mobile phone use > 10 years was linked to higher risk of all types of intracranial tumors. Wang and Guo also in their recent meta-analysis reported a significant association between mobile phone use for more than 5 years and the risk of glioma (Wang and Guo, 2016).

Moreover, the findings of a recent large-scale study conducted by the U.S. National Toxicology Program (NTP) revealed statistically significant increases in cancer in rodents that had been exposed to GSM or CDMA signals for 2-years. NTP study showed that when the intensity of the radiation increased, the incidence of cancer among the rats also increased (Wyde et al., in review). This 25,000,000 USD study that is the most complex study completed by the NTP, showed that the occurrence of malignant gliomas in the brain and schwannomas of the heart, can be linked to exposure to mobile phone radiofrequency radiation (RFR) “*The occurrences of two tumor types in male Harlan Sprague Dawley rats exposed to RFR, malignant gliomas in the brain and schwannomas of the heart, were considered of particular interest, and are the subject of this report*.”

It should be noted that recently Momoli et al. (2017) have performed a re-analysis of the Canadian data from the 13-country INTERPHONE case-control study and when they applied a probabilistic multiple-bias model to address possible biases simultaneously, the odds ratio (OR) for glioma comparing highest quartile of use (>558 cumulative lifetime hours of use) to non-regular users was 2.0 (95% confidence interval: 1.2, 3.4). When adjusted for selection and recall biases, the OR was 2.2 (95% confidence interval: 1.3, 4.1).

## Author contributions

The author confirms being the sole contributor of this work and approved it for publication.

### Conflict of interest statement

The author declares that the research was conducted in the absence of any commercial or financial relationships that could be construed as a potential conflict of interest.
